# Drug reaction with eosinophilia and systemic symptoms (DRESS) syndrome after topical use of *Nigella sativa* (black cumin) oil

**DOI:** 10.1111/cod.14127

**Published:** 2022-05-30

**Authors:** Marine Fargeas, Andreea Calugareanu, Benoit Ben‐Said

**Affiliations:** ^1^ Severe Cutaneous Adverse Drug Reaction Regional Center, Department of Dermatology Hospices civiles de Lyon Lyon France

**Keywords:** black cumin, case report, drug reaction, drug reaction with eosinophilia and systemic symptoms (DRESS), *Nigella sativa*

*Nigella sativa*, also known as ‘black cumin’, belongs to the family of Ranunculaceae and is mainly used in the form of oil.^1^ The main constituent of the *Nigella Sativa* oil (NSO) is thymoquinone, which represents between 27.8 and 57% of the essential oil. NSO is the subject of studies for its pharmacological properties in various fields such as oncology^2^ and allergology.^3^ Few case reports of contact dermatitis,^4^, ^5^, ^6^ bullous delayed hypersensitivity and Stevens–Johnson syndrome or toxic epidermal necrosis related to NSO have been published.^7^, ^8^ To date, only one case of drug reaction with eosinophilia and systemic symptoms (DRESS), a delayed T‐cell adverse drug reaction, after application of NSO has been reported.^9^ We report here a case of DRESS after application of NSO confirmed by positive patch test.

## CASE REPORT

**FIGURE 1:**
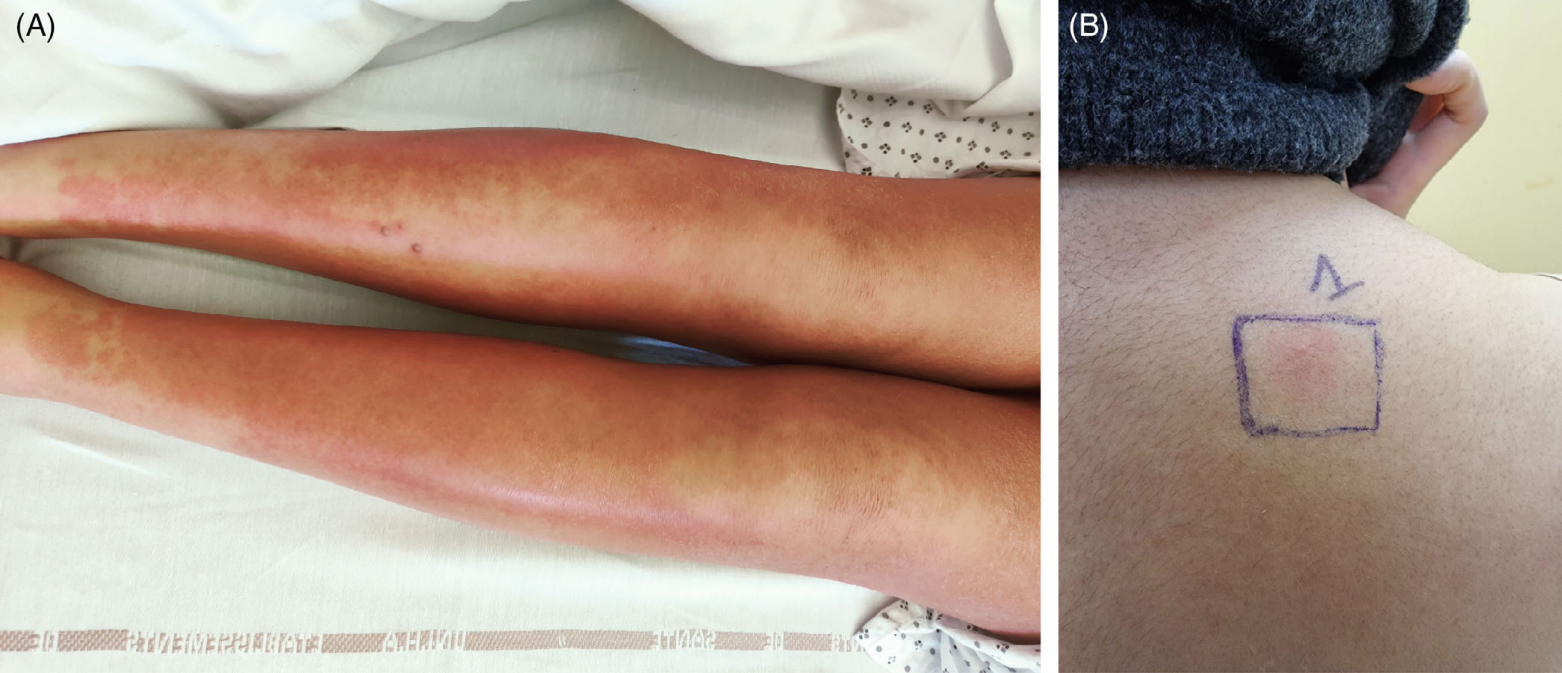
Generalized, infiltrated, pruritic maculopapular exanthema (A); positive epicutaneous patch test with black cumin oil brought by the patient (concentration of *Nigella sativa*: 0.001%) at day(D) 3 reading (B)

A 28‐year‐old woman, with no medical history and no history of allergies, presented with generalized exanthema sparing the face 1 week after application of NSO on the whole tegument including the face once daily. Clinical examination at the admission revealed extensive and infiltrative exanthema associated with oedema of extremities and enlarged lymph nodes. There was no mucosal involvement and no Nikolsky sign. Blood tests showed eosinophilia at 2.89*10^9^/L (N < 0.5*10^9^/l) and atypical lymphocytes. There was no visceral involvement, especially no kidney or liver failure. Epstein–Barr virus (EBV), cytomegalovirus (CMV), parvovirus B19, human herpes virus 6 (HHV6), human herpes virus 8 (HHV8) blood PCR viral load remained negative. Histologic analysis of a 4‐mm lesional skin biopsy of the upper thigh showed eosinophilic spongiosis without lichenoid alterations or keratinocyte necrosis, as well as an intense superficial lymphocytes and eosinophils perivascular infiltrate. Direct immunofluorescence was negative. The Kardaun severity score was evaluated at 5 (skin rash suggesting DRESS +1, skin rash affecting >50% of the body surface area + 1, eosinophilia >1.5 g/L + 2, atypical lymphocytes +1, enlarged lymph nodes +1, no fever ‐1)^10^ suggesting the diagnosis of DRESS induced by NSO. Systemic corticosteroids (40 mg/day prednisone) were initiated and resulted in complete regression of the exanthema within 1 month. Six months later epicutaneous patch test were performed with NSO brought by the patient and tested ‘as is’ with Finn Chamber (Epitest, Antony, France) (concentration of *Nigella sativa*: 0.001%): at day(D) 3 (after 2 days of occlusion) reading was positive with infiltration and a discrete papule (+ according to International Contact Dermatitis Research Group, ICDRG, criteria; as seen in Figure 1). Patch test with the same oil performed on three team members' as negative controls (with their agreement) remained negative.

## DISCUSSION

We report here a case of DRESS syndrome after application of NSO confirming the associated risk of severe cutaneous adverse reactions (SCARS). The attributed therapeutic properties, the consumers' attraction to ‘biologic’ or natural products and the easy access as over‐the‐counter product may lead to underestimation of the potential side effects and increased use of NSO in the future.^11^ Therefore, clinicians should be aware of SCARS related to NSO and inform their patients about this risk. Moreover, SCARS related to NSO should be reported to the pharmacovigilance network.

## FUNDING SOURCES

This article has no funding source.

## CONFLICTS OF INTEREST

The authors have no conflict of interest to declare.
